# Stress in dermatology patients: A multicenter observational study of 8295 outpatients and controls from 22 European clinics

**DOI:** 10.1016/j.jdin.2025.12.005

**Published:** 2025-12-26

**Authors:** Flora Balieva, Christina Schut, Csanád Szabó, Francesca Sampogna, Florence J. Dalgard, Ilknur K. Altunay, Anthony Bewley, Bárbara Roque Ferreira, Andrew Y. Finlay, Uwe Gieler, Tamara Gracia-Cazaña, Vesna Grivcheva-Panovska, Gregor B. Jemec, Franz J. Legat, Lars Lien, Andrey Lvov, Servando E. Marron, Laurent Misery, Adam Reich, Dmitry Romanov, Saskia Spillekom-van Koulil, Sonja Ständer, Ake Svensson, Jacek C. Szepietowski, Andrew R. Thompson, Geraldine Titeca, Lucía Tomás-Aragonés, Nienke Vulink, Claudia Zeidler, Jörg Kupfer, ESDaP Study collaborators

**Affiliations:** a Department of Dermatology, Stavanger University Hospital, Stavanger, Norway; b Department of Clinical Medicine, University of Bergen, Bergen, Norway; c Institute of Medical Psychology, Justus-Liebig-University, Gießen, Germany; d Department of Police, University of Applied Sciences for Public Management and Security, Wiesbaden, Germany; e Institute of Psychology, University of Szeged, Szeged, Hungary; f Department of Dermatology and Allergology, University of Szeged, Szeged, Hungary; g Clinical Epidemiology Unit, Istituto Dermopatico dell’Immacolata (IDI)-IRCCS, Rome, Italy; h Department of Psychiatry, Lovisenberg Diaconal Hospital, Oslo, Norway; i Department of Dermatology and Venereology, Skåne University Hospital, Malmo, Sweden; j Department of Dermatology, University of Health Sciences, Hamidiye Etfal Training and Research Hospital, Istanbul, Turkey; k Barts Health NHS Trust & Queen Mary University of London, London, UK; l Department of Dermatology, Coimbra Hospital and University Centre, Coimbra, Portugal; m Faculty of Medicine and Biomedical Sciences, University of Algarve, Faro, Portugal; n Division of Infection and Immunity, School of Medicine, Cardiff University, Cardiff, UK; o Deptartment of Dermatology, University Gießen Germany; p Department of Dermatology, University Hospital Miguel Servet, Zaragoza, Spain; q University St Cyril and Methodius, School of Medicine, PHI University Clinic of Dermatology, Skopje, North Macedonia; r Department of Dermatology, PHOENIX Center, Herlev-Gentofte Hospital, Gentofte, Denmark; s Faculty of Health and Medical Sciences, University of Copenhagen, Copenhagen, Denmark; t Department of Dermatology, Medical University of Graz, Graz, Austria; u Faculty of Social and Health Sciences, Inland Norway University of Applied Sciences, Elverum, Norway; v Norwegian National Advisory Unit on Concurrent Substance Abuse and Mental Health Disorders, Innlandet Hospital Trust, Brumunddal, Norway; w Central State Medical Academy of Department of Presidential Affairs, Moscow, Russia; x Medical Research and Educational Centre, Lomonosov Moscow State University, Moscow, Russia; y Aragon Psychodermatology Research Group Zaragoza, Zaragoza, Spain; z Department of Dermatology, University Hospital Miguel Servet, Zaragoza, Spain; aa Department of Dermatology, University Hospital of Brest, Brest, France; ab Department of Dermatology, Faculty of Medicine, Medical College of Rzeszów University, Rzeszów, Poland; ac Department of Psychiatry and Psychosomatics, I.M. Sechenov First Moscow State Medical University (Sechenov University), Moscow, Russia; ad Department of Boundary Mental Conditions and Psychosomatic Disorders, Mental Health Research Centre, Moscow, Russia; ae Department of Medical Psychology, Radboud Institute for Health Sciences, Radboud University Medical Centre, Nijmegen, the Netherlands; af Pruritus Medicine Section, Department of Dermatology and Center for Chronic Pruritus (KCP), University Hospital Münster, Münster, Germany; ag Division of Dermatology, Venereology and Clinical Immunology, Faculty of Medicine, Wroclaw University of Science and Technology, Wroclaw, Poland; ah Department of Dermato-Venereology, 4th Military Hospital, Wroclaw, Poland; ai South Wales Clinical Psychology Training Programme, Cardiff & Vale University Health Board & School of Psychology, Cardiff University, Cardiff, UK; aj Clinique Notre Dame de Grace, Gosselies, Belgium; ak Department of Psychology, University of Zaragoza, Zaragoza, Spain; al Department of Psychiatry, Amsterdam UMC, University of Amsterdam, Amsterdam, the Netherlands

**Keywords:** anxiety, body dysmorphic disorder, depression, dermatological disease, epidemiology, European, multicenter, observational case-controlled study, outpatients, PSS-10, psychosocial burden, stigmatization, stress

## Abstract

**Background:**

Skin diseases are symptomatic, visible, and stigmatizing and it is acknowledged that they can be associated with stress. However, large studies comparing disease-specific stress are scarce.

**Objectives:**

To investigate stress in a large, diverse sample of patients with different skin conditions and identify predictors of stress.

**Methods:**

A cross-sectional, multicenter study was conducted in 22 dermatology clinics across 17 European countries (response rate 82.4%). The study included 5487 patients diagnosed with various dermatological conditions and 2808 skin-healthy controls. The Perceived Stress Scale, 10 items was used to measure stress.

**Results:**

Patients reported significantly higher stress levels, more stressful life events during the last 6 months, and more economic difficulties than controls. Patients with psychodermatological conditions, hyperhidrosis, hidradenitis suppurativa, atopic dermatitis, acne, and urticaria experienced the highest stress levels. 44% of the variance of perceived stress in patients with skin conditions could be predicted by sociodemographic data and disease-related and psychological variables (depression, anxiety, stigmatization, and body dysmorphic concerns).

**Limitations:**

As with all cross-sectional studies, causality and directionality cannot be inferred.

**Conclusion:**

Stress poses a significant psychosocial burden to dermatological patients, especially to vulnerable subgroups. Health interventions targeting stress may be essential to improve clinical outcomes.

**Capsule Summary**

• Patients with various skin diseases experience greater stress and psychological burden compared with skin-healthy controls.
• Integrating stress assessment and psychosocial care particularly in inflammatory or stigmatizing dermatologic conditions may improve clinical outcomes.

## Introduction

Stress is a state of real or perceived threat to homeostasis, eliciting responses involving the nervous, endocrine, and immune systems.^1^ Chronic stress is defined as the repeated occurrence of various stressors with uncontrollable consequences over a sustained period in the absence of adaptive coping mechanisms.^2^ While stressors such as illness, interpersonal conflict, or financial strain are common, individuals differ significantly in how they perceive and respond to these challenges.^3^

In the context of chronic disease, stress can interfere with disease management. Conversely, chronic illness may serve as an ongoing stressor, particularly when symptoms are visible or stigmatizing, as in dermatological conditions.^3^, ^4^, ^5^

Studies have shown elevated stress levels among patients with psoriasis, atopic dermatitis (AD), chronic urticaria, acne, and other skin diseases.^5^, ^6^, ^7^ These patients frequently also report high levels of anxiety, depression, social withdrawal, and impaired sleep, which may further exacerbate disease severity through psychoneuroimmunological mechanisms. Other studies^6^^,^^8^, ^9^, ^10^, ^11^ support a bidirectional relationship between stress and skin diseases, where stress may worsen inflammation and the dermatological symptoms, while visible lesions and discomfort may increase the emotional burden.^7^^,^^8^^,^^12^ Previous research indicates that stress and mental health disorders can result from skin diseases and also worsen them.^9^

Zhang et al^13^ describe the neuroendocrine-immune mechanisms by which stress acts as a crucial factor in the development of several skin diseases^13^ in which stress plays a significant role by triggering the release of inflammatory cytokines, which in turn worsens inflammation and further exacerbates the inflammatory skin disease.^13^

Misery et al^6^ examined perceived stress, quality of life, and self-reported disease severity in 7273 adults with acne, AD, psoriasis, and hidradenitis suppurativa (HS). Stress levels were very high across all conditions. Higher disease severity correlated with higher perceived stress and severe stress was registered in 70.4% of acne patients, 66.3% of psoriasis patients, 65.3% of AD patients, and 52.8% of HS patients.^6^

Risk factors influencing stress and coping mechanisms may be sociodemographics (age, sex,^14^ and income) or factors such as comorbidity,^7^ disease severity,^6^ itch,^15^ stigmatization,^16^, ^17^, ^18^ and body dysmorphic concerns (BDC)/body dysmorphic disorder (BDD).^18^^,^^19^ The meta-analysis conducted by Salari et al^9^ confirmed a link between skin diseases and mental health issues, with the highest rate of stress observed in patients with acne (75.7%).

Despite growing recognition of the role of psychological factors in dermatology, previous studies of stress in skin diseases have often been limited by small sample sizes, narrow diagnostic focus, or the absence of appropriate control groups, making it difficult to draw conclusions about the overall burden of stress and its relationship with other psychosocial variables in dermatological patients.

As a result, there is a need for larger, controlled studies involving broader patient populations to better understand the extent of stress experienced by dermatological patients and how it relates to common comorbidities such as anxiety and depression.

To address this gap, the present study examines a large sample of dermatological outpatients to investigate whether patients with skin conditions differ from skin-healthy controls in terms of stress as measured by the Perceived Stress Scale (PSS), 10 items and their reported economic difficulties and stressful life events. In addition, we investigate whether certain groups of dermatological patients are more affected by stress than others. Finally, the study examines whether stress is related to sociodemographic, disease-related, and psychological variables (depression, anxiety, stigmatization, and BDC/BDD) across diagnostic groups.

By providing a broader understanding of stress in dermatological populations, this study may help to identify subgroups which might benefit from psychosocial support as part of their treatment.

## Methods

### Study design, ethics, participants, and procedure

This observational cross-sectional multicenter study included dermatology outpatients and controls from 22 dermatology clinics in 17 European countries treated between September 2017 and December 2019. It was approved by the Institutional Review Board of the Department of Medicine at the University of Gieβen (protocol number 87/17) and at each recruitment center. The study was registered at the German Registry for clinical studies (Registration number: DRKS00012745) and conducted in concordance with the Declaration of Helsinki.^20^

Each site recruited 250 consecutive, adult dermatological outpatients and 125 skin-healthy controls. Controls with healthy skin were recruited by inviting hospital staff and visitors to participate, for example, using written notices and announcements at staff meetings. They were excluded if they had any skin condition. Study participants had to be at least 18 years old and able to read and understand the questionnaires as well as able to give consent. All participants signed a written, informed consent before inclusion.^19^

The current paper analyzes data from a large study (the second project of the European Society for Dermatology and Psychiatry [European Society for Dermatology and Psychiatry study II]) designed to explore multiple aspects of psychological burden in dermatological patients. The large dataset collected allows for various analyses regarding psychodermatological issues, and the present paper addresses the stress component, while other publications have focused, or will focus, on other psychological outcomes from the same dataset.^14^, ^15^, ^16^^,^^18^^,^^19^

### Variables

#### Sociodemographic and general health-related variables

Age, sex, and income level were self-reported. Physical comorbidities (diabetes, cardiovascular, chronic respiratory, rheumatological disease, or other) were recorded by the dermatologist, while controls self-reported their comorbidities and were excluded if they reported having a skin disease.

#### Disease-related variables

Clinical assessment was performed by the dermatologist, who classified the skin condition according to International Classification of Diseases, 10th Revision criteria with a precision of at least 1 numeral after the digit.^19^ Disease severity was rated as ‘mild’, ‘moderate’, or ‘severe’ by the dermatologist. The prevalence of itch was investigated by the question: ‘Have you suffered from itch in the last 24 hours?’ (yes/no).

#### Stress

Perceived stress was measured using the PSS-10, a 10-item scale (range: 0-40) that assesses the degree to which individuals perceive their lives as unpredictable, uncontrollable, and overloading when external demands are judged to have exceeded their ability to cope during the previous month.^21^^,^^22^ Contrary to measures which assess if stressful life events occurred, the PSS measures the degree to which situations in one's life are appraised as stressful.^21^, ^22^, ^23^ One dimension is related to ‘perceived helplessness’, another to ‘reduced perceived self-efficacy’.^24^ PSS has demonstrated good reliability and stability over time.^25^

The occurrence of stressful life events and economic difficulties were evaluated by asking if the participant had experienced stressful life events and economic difficulties during the last 6 months (yes/no).

#### Psychological variables

Presence of depression and anxiety was screened using the Patient-Health-Questionnaire 2-item and the General-Anxiety-Disorder 2-item^26^ using a cut-off score of ≥3 for screened depression or anxiety.

#### Perceived stigmatization

Perceived stigmatization was assessed with the 21-item Perceived Stigmatization Questionnaire (PSQ),^27^ consisting of 3 subscales: ‘absence of friendly behavior’, ‘confused/staring behavior’, and ‘hostile behavior’. This questionnaire can be used across different health conditions.^28^, ^29^, ^30^ Items are answered on a 5-point Likert scale (0-4 points), with higher scores reflecting higher levels of perceived stigmatization. PSQ was designed to sample stigmatizing behaviors commonly reported by people with appearance distinctions.^27^ Low, medium, and high perceived stigmatization were defined based on terciles of the distribution of PSQ total score.

#### Self-assessed HRQoL

Participants rated their health state on a visual analog scale from zero (worst imaginable) to 100 (best imaginable), using the European quality of life 5 dimension, Visual Analog Scale.^31^

#### Body dysmorphic disorder/body dysmorphic concerns

BDD/BDC symptoms were measured using a validated self-reported screening instrument for Diagnostic and Statistical Manual of Mental Disorders, Fourth Edition criteria, the Dysmorphic Concern Questionnaire,^32^ which measures concerns about one’s own body appearance. The instrument has 7 items which are answered on a scale from 0 to 3. Total scores range from 0 to 21.

Extensive information on these instruments is given in the study protocol.^20^

### Statistical analysis

Differences between patients and controls in the sociodemographic variables were tested using Chi-square tests, *t*-tests, or analyses of variance.

*T*-tests were performed to determine whether patients with different skin conditions and skin-healthy controls differed regarding stress (PSS-10 total score, PSS score for helplessness, and PSS score for self-efficacy). In addition, Chi-square tests were conducted to compare the occurrence of economic difficulties and stressful life events during the last 6 months between patients with different skin conditions and skin-healthy controls.

A linear regression model including the different skin diseases was performed to investigate whether perceived stress could be predicted by sociodemographic variables (sex, age, income; step 1), disease-related variables (itch, comorbidities, severity of the skin disease, general health status; step 2), and psychological variables (screened depression, screened anxiety, screened BDD/BDC, perceived stigmatization: absence of friendly behavior, confused behavior by others and hostile behavior by others; step 3).

Continuous variables were described with mean and standard deviation (±standard deviation) and categorical variables with number and percentage.

## Results

### Sample characteristics

A total of 5487 dermatological outpatients (response rate 82.4%) and 2808 healthy controls were included in the study. Among patients, *n* = 5265, and among controls, *n* = 2748, gave valid responses to the PSS items used to construct scales. As shown in Table I, there were significant differences (*P* < .001) between patients and controls in all measured variables (sex, age, income, comorbidities, and itch). A large proportion of patients (65%) had a moderate to severe skin disease.

**Table I:** Sample characteristics

Variable	Patients (n = 5265)	Controls (n = 2748)	*P* value
Sex, *n* (%)			<.001
Male	2251 (43.1%)	904 (33.0%)	
Female	2976 (56.9%)	1837 (67.0%)	
Age, mean (±standard deviation), y	48.3 (17.5)	42.8 (15.5)	<.001
Income, *n* (%)			<.001
Low	1438 (28.2%)	504 (18.6%)	
Middle	3188 (62.6%)	1763 (64.9%)	
High	470 (9.2%)	448 (16.5%)	
Comorbidities, *n* (%)			<.001
No	2724 (52.8%)	1946 (72.9%)	
Yes	2433 (47.2%)	724 (27.1%)	
Itch, *n* (%)			<.001
No	1995 (38.3%)	2306 (84.6%)	
Yes	3216 (61.7%)	420 (15.4%)	
Clinical severity, *n* (%)			
Mild	1772 (35.2)	-	-
Moderate	2291 (45.5)		
Severe	976 (19.4)		

Description of the 8013 participants whose data were used in the further analyses.

Females (56.9%) had stress levels significantly higher compared to males (mean [standard deviation]: 17.3 [7.0] vs 14.6 [6.9], *P* < .001). Likewise, younger participants and those with lower income reported significantly higher stress levels (*P* < .001). Clinical factors associated with higher perceived stress included the presence of comorbidities, moderate to severe disease severity, and chronic itch (*P* < .001). Patients with depression, anxiety, or BDD/BDC reported markedly higher stress levels than those without these conditions (Table II).

**Table II:** Mean Perceived Stress Scale (PSS) score with standard deviation (SD) according to different sociodemographic and clinical variables

Variable	*N*∗ (%)	PSS total mean (±SD)	*P* value†
Sex			
Male	2251 (43.1)	14.9 (6.9)	<.001
Female	2976 (56.9)	17.3 (7.0)	
Age (terciles, y)			
<39	1723 (33.0)	17.4 (6.8)	<.001
39-57	1771 (34.0)	16.8 (7.2)	
>57	1723 (33.0)	14.6 (6.9)	
Income			
Low	1438 (28.2)	18.4 (7.2)	<.001
Middle	3188 (62.6)	15.7 (6.8)	
High	470 (9.2)	13.7 (6.6)	
Itch			
None	1995 (40.0)	14.6 (6.8)	<.001
Acute	1089 (21.8)	17.1 (6.5)	
Chronic	1906 (38.2)	17.6 (7.3)	
Comorbidities			
No	2724 (52.8)	16.0 (6.8)	<.001
Yes	2433 (47.2)	16.6 (7.3)	
Clinical severity			
Mild	1772 (35.2)	15.3 (6.7)	<.001
Moderate	2291 (45.5)	16.5 (6.8)	
Severe	979 (19.4)	17.7 (7.9)	
Depression			
No	3859 (74.6)	14.4 (6.3)	<.001
Yes	1311 (25.4)	21.9 (6.3)	
Anxiety			
No	3810 (73.6)	14.1 (6.1)	<.001
Yes	1364 (26.4)	22.4 (5.9)	
Screened BDD/BDC			
No	4650 (89.4)	15.5 (6.7)	<.001
Yes	550 (10.6)	22.6 (6.6)	

*BDD/BDC*, Body dysmorphic disease/body dysmorphic concerns; *PSS*, Perceived Stress Scale; *SD*, standard deviation.

∗ Totals may vary because of missing values.

† From *t*-test or ANOVA.

Stress levels varied substantially across diagnostic groups (Table III). Patients with psychodermatological conditions, hyperhidrosis, HS, AD, acne, and urticaria reported the highest PSS scores (PSS range: 17.9-21.2). These groups also showed high scores of perceived helplessness and reduced self-efficacy. In contrast, patients with nonmelanoma skin cancer (NMSC), malignant melanoma (MM), and melanocytic nevi had the lowest PSS scores (12.7-13.7).

**Table III:** Mean and standard deviation (± SD) for Perceived Stress Scale (PSS) total score, PSS-perceived helplessness and PSS-reduced perceived self-efficacy for all skin disease groups and significance compared with controls

Diagnosis (*n* = 8013)	PSS total mean (±SD)	PSS-perceived helplessness mean (±SD)	PSS-reduced perceived self-efficacy mean (±SD)
**Controls (2748)**	14.3 (6.3)	9.0 (4.8)	5.3 (3.1)
**Patients (5265)**	16.3 (7.1)∗	10.2 (5.4)∗	6.1 (3.3)∗
**Psychodermatological conditions**†**(18)**	21.2 (7.9)∗	14.3 (6.4)‡	6.9 (3.2)
**Hyperhidrosis (25)**	20.5 (8.2)∗	12.9 (5.4)§	7.7 (3.7)§
**Hidradenitis suppurativa (136)**	19.5 (7.3)∗	12.5 (5.6)∗	7.0 (3.0)∗
**Atopic dermatitis (344)**	18.1 (6.5)∗	11.9 (5.0)∗	6.1 (2.6)∗
**Acne (231)**	18.0 (6.6)∗	11.5 (5.0)∗	6.5 (3.1)∗
**Urticaria (168)**	17.9 (6.7)∗	11.7 (4.8)∗	6.2 (2.8)§
**Allergies/hypersensitivity reactions (114)**	17.6 (5.5)∗	11.4 (4.5)∗	6.2 (3.3)‡
**Alopecia areata (83)**	17.5 (7.6)§	11.3 (5.3)∗	6.2 (3.2)
**Psoriasis (1380)**	17.0 (7.2)∗	10.8 (5.5)∗	6.2 (3.4)∗
**Chronic prurigo (124)**	16.8 (6.7)∗	11.0 (5.0)∗	5.8 (3.0)
**Rosacea (86)**	16.6 (6.3)‡	10.5 (5.4)	6.0 (3.4)
Connective tissue disease (214)	16.5 (6.0)∗	10.2 (4.9)‡	6.3 (3.4)∗
Papulosquamous disorders‖ (117)	16.3 (7.2)‡	10.4 (5.4)‡	5.9 (3.1)
Bullous diseases (138)	16.2 (7.1)§	9.8 (5.6)	6.4 (3.7)§
Pruritus (106)	16.1 (6.3)‡	10.2 (5.0)	6.0 (3.1)
Dermatitis, not otherwise specified¶ (243)	15.8 (7.3)‡	10.0 (5.6)	5.9 (3.3)
Vitiligo (46)	16.9 (7.8)	11.0 (5.9)	5.9 (3.5)
Venous insufficiency (73)	16.1 (7.5)	10.1 (5.8)	6.0 (3.4)
Seborrheic dermatitis (58)	15.9 (7.1)	10.1 (5.0)	5.8 (3.3)
Hand eczema (71)	15.8 (5.9)	10.1 (4.5)	5.7 (2.9)
Alopecias, other than alopecia areata (64)	15.8 (6.1)	9.7 (4.6)	6.1 (2.8)
Infections (240)	15.5 (6.9)	9.7 (5.3)	5.8 (3.3)
Metabolic/systemic diseases (102)	15.5 (7.4)	9.7 (5.5)	5.9 (3.4)
Benign tumors# (212)	15.0 (6.7)	9.1 (5.1)	6.0 (3.6)
Skin malformations∗∗ (53)	14.7 (7.0)	8.8 (4.8)	5.9 (3.5)
Melanocytic nevi (225)	13.7 (6.6)	8.3 (5.1)	5.4 (3.6)
Malignant melanoma (95)	12.9 (7.2)	7.6 (5.2)	5.2 (3.6)
**Nonmelanoma skin cancer (440)**	12.7 (6.8)∗	7.0 (5.1)∗	5.7 (3.9)
Others†† (59)	15.5 (6.2)	9.3 (4.9)	6.2 (3.4)

Conditions with significant values are highlighted in bold.

*P* values from *t*-tests are corrected with Holm-Bonferroni method for multiple comparisons.

*PSS*, Perceived Stress Scale; *SD*, standard deviation.

∗ *P* < .001.

† Paranoid psychosis, obsessive compulsive disorder, somatoform disease, trichotillomania, hallucinations.

‡ *P* < .05.

§ *P* < .01.

‖ Excluding psoriasis.

¶ Excluding atopic dermatitis, hand eczema, and seborrheic dermatitis.

# Lipomas, hemangiomas, granulomas, tumors of the connective and soft tissue (excluding melanocytic nevi), benign cysts, seborrheic keratosis, cutaneous horn, hypertrophic scars, pyogenic granuloma.

∗∗ Vascular malformations, epidermal nevi, ichthyosis, neurofibromatosis.

†† Undefined tumor, disease of the oral mucosa, nail disorders, balanoposthitis, burns/injuries, observation.

### Comparison between patients and skin-healthy controls

**Fig 1:**
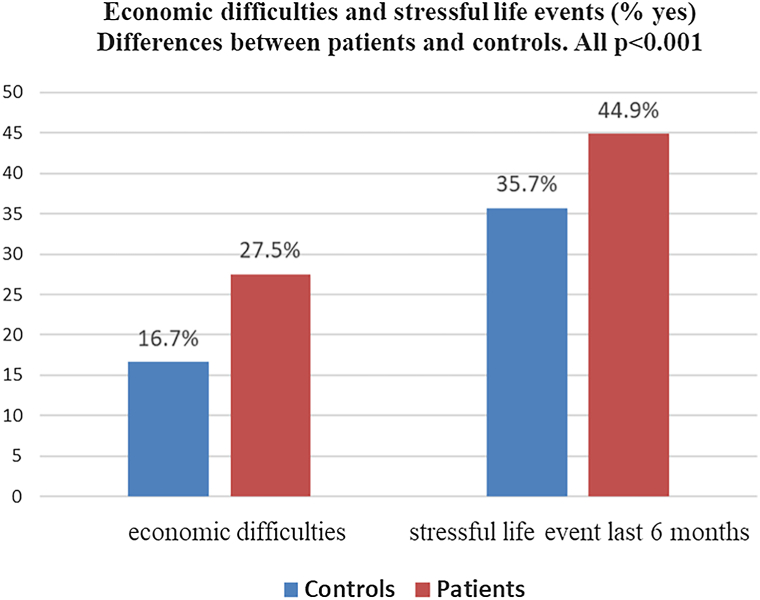
Economic difficulties and stressful life events in the last 6 months.

Patients with dermatological conditions reported significantly higher total stress scores than controls (PSS mean: 16.3 vs 14.3, *P* < .001). This difference was evident for both PSS subscales—perceived helplessness (10.2 vs 9.0, *P* < .001) and reduced perceived self-efficacy (6.1 vs 5.3, *P* < .001) (Table III). In addition, dermatological patients were more likely to report stressful life events and economic difficulties within the past 6 months (Fig 1), further confirming a higher overall burden of stress in this population.

### Sociodemographic, clinical, and psychological variables as predictors of stress in dermatological patients

Psychological factors were significantly related to stress in the hierarchical regression analysis, while sociodemographic variables (age, sex, income) explained 8.5% of the variance in PSS scores in the stepwise modeling. Adding disease-related variables (itch, comorbidities, severity, European quality of life 5 dimension, Visual Analog Scale health status) increased the explained variance to 24.2%. The inclusion of psychological variables (depression, anxiety, BDD/BDC, perceived stigmatization) yielded an adjusted R^2^ = 0.44 (Table IV), indicative of a high goodness-of-fit.^33^ Anxiety (B = 4.13, *P* < .001), depression (B = 2.74, *P* < .001), BDD/BDC (B = 1.76, *P* < .001), and all perceived stigmatization categories were significantly associated with elevated stress levels. Notably, perceived hostile behavior from others showed the strongest association with perceived stress among the stigmatization subscales (B = 0.20, *P* < .001).

**Table IV:** Linear regression model with Perceived Stress Scale (PSS) total score as dependent variable: PSS in dermatological patients is associated with sex, age, income, comorbidities, disease severity, EQ-VAS health status, screened depression, anxiety, and body dysmorphic disease/concerns and all stigmatization subgroups

	Unstandardized B (95% CI)	Unstandardized B (95% CI)	Unstandardized B (95% CI)
	Step 1	Step 2	Step 3
Constant	20.42 (19.27; 21.58)∗	27.91 (26.47; 29.36)∗	18.33 (16.96; 19.71)∗
Sex (female vs male)	2.06 (1.64; 2.48)∗	1.76 (1.38; 2.14)∗	1.40 (1.07; 1.73)∗
Age (continuous)	−0.07 (−0.08; −0.06)∗	−0.08 (−0.10; −0.07)∗	−0.05 (0.06; −0.04)∗
Income (low, middle, high)	−2.32 (−2.67; −1.97)∗	−1.53 (−1.85; −1.21)∗	−0.85 (−1.13; −0.57)∗
Step 2			
Itch (yes vs no)		0.64 (0.42; 0.86)∗	0.16 (−0.03; 0.35)
Comorbidities		0.66 (0.24; 1.07)†	0.47 (0.12; 0.83)†
Disease severity		0.03 (−0.24; 0.31)	−0.37 (−0.61; −0.13)†
EQ-VAS health status		−0.13 (−0.14; −0.12)∗	−0.06 (−0.07; −0.05)∗
Step 3			
Screened depression			2.74 (2.29; 3.20)∗
Screened anxiety			4.13 (3.68; 4.58)∗
Screened BDD/BDC			1.76 (1.19; 2.33)∗
PSQ–absence of friendly behavior			0.11 (0.09; 0.14)∗
PSQ–confused behavior			0.11 (0.08; 0.14)∗
PSQ–hostile behavior			0.20 (0.14; 0.27)∗
Adjusted R^2^	0.085	0.242	0.440

The adjusted R^2^ for the overall model was 0.44, indicative of a high goodness-of-fit. Sociodemographic, disease-related, and psychological variables were able to statistically significantly predict the value of the PSS total scale, F (13, 4162) = 253.73, *P* < .001.

*BDD/BDC*, Body dysmorphic disorder/body dysmorphic concerns; *CI*, confidence interval; *EQ-VAS*, EuroQol5D Visual Analog Scale; *PSQ*, Perceived Stigmatization Questionnaire.

∗ *P* < .001.

† *P* < .01.

## Discussion

This large multicenter study confirms that dermatological patients experience significantly higher psychological stress than individuals with healthy skin. Across the total sample, stress was more prevalent in women, younger participants, individuals with lower income, and those reporting comorbidities or more severe disease. Importantly, stress was most strongly associated with psychological comorbidities (depression, anxiety, BDC, and perceived stigmatization).

The PSS allows for a nuanced understanding of stress appraisal by distinguishing between perceived helplessness and reduced self-efficacy.^24^^,^^25^ Both dimensions were higher in patients compared to controls, indicating not only higher emotional burden but also a reduced ability to cope. This supports theories that chronic, visible, or stigmatizing illness may impair one’s sense of control and amplify distress.^3^^,^^4^

Our findings are in line with previous research showing elevated stress in dermatological populations.^6^^,^^9^ Misery et al^6^ reported very high stress levels in patients with acne, AD, psoriasis, and HS, as in our sample. However, our study, besides having a control group, also showed perceived helplessness and reduced self-efficacy in these conditions and in psychodermatological conditions, hyperhidrosis, urticaria, allergies, chronic prurigo, and connective tissue diseases. The results underscore the vulnerability of several other dermatological conditions, not yet explored.

The role of stigmatization in stress was clearly demonstrated. All 3 subscales of perceived stigmatization were significantly associated with higher stress scores, especially perceived hostile behavior. This aligns with previous research which indicated that stigmatization contributes to psychological distress and social withdrawal in dermatological populations.^16^^,^^17^

While sociodemographic and disease-related factors explained some of the variance in stress, psychological variables, especially anxiety and depression, accounted for the largest share. This highlights the importance of integrated care models which address not only the physical but also the emotional and social dimensions of skin disease.

Not all skin conditions were associated with elevated stress levels. Patients with solitary, noninflammatory skin conditions (NMSC, MM, and melanocytic nevi) showed lower perceived stress than the controls (eg, NMSC, *P* < .001). Patients with NMSC and MM were significantly older than the controls which may partly explain lower stress. The availability of effective curative treatments and lack of chronic inflammation may also be important. Several previous studies have likewise shown lower disease burden in patients with solitary lesions compared to patients with inflammatory skin conditions.^34^^,^^35^

Stress may be important in triggering cutaneous inflammation and several recent epidemiological studies demonstrate that stress often predates the onset of skin disease in subsets of patients.^7^^,^^10^^,^^13^ Our study indicates an association between stress, mental health symptoms, and dermatological conditions and supports a bidirectional relationship, consistent with some previous studies and biological neuroendocrine-immune models of skin–brain interaction.^7^^,^^13^

### Strengths and limitations

A key strength of this study is its large, diverse sample and inclusion of a skin-healthy control group, which improves generalizability. By identifying subgroups at particular risk, such as those with psychodermatological conditions, hyperhidrosis, HS, AD, acne, and urticaria, the findings provide targets for intervention. Although the PSS does not have validated subscores or established clinical cut-offs, the consistent and statistically significant differences observed support a higher stress burden among dermatological patients. The large sample size is likely to have contributed to detecting statistically significant differences, although the mean differences were rather small.

The cross-sectional design limits causal inference on whether stress exacerbates skin disease or vice versa. Self-reported measures may be affected by recall or response bias. The patient and control populations differed significantly in several baseline characteristics, which represent a potential source of bias. The PSS is a well-validated tool; however, it does not capture the type or duration of stressors. Moreover, the sample size for some patient groups was rather small.

## Conclusion

These findings reinforce the need for dermatologists to be aware of general stress experience and psychological comorbidity in their patients. Particular attention should be paid to patients with high-risk conditions, such as stigmatizing experiences, BDD/BDC, or co-occurring depression and anxiety. Psychosocial interventions, stress management techniques, and referral to mental health services when integrated into dermatological care are likely to improve clinical outcomes, especially for patients reporting reduced self-efficacy or perceived helplessness.

## Conflicts of interest

None disclosed.
